# Molecular Transducers from Roots Are Triggered in Arabidopsis Leaves by Root-Knot Nematodes for Successful Feeding Site Formation: A Conserved Post-Embryogenic *De novo* Organogenesis Program?

**DOI:** 10.3389/fpls.2017.00875

**Published:** 2017-05-26

**Authors:** Rocío Olmo, Javier Cabrera, Miguel A. Moreno-Risueno, Hidehiro Fukaki, Carmen Fenoll, Carolina Escobar

**Affiliations:** ^1^Área de Fisiología Vegetal, Facultad de Ciencias Ambientales y Bioquímica, Universidad de Castilla La ManchaToledo, Spain; ^2^Centro de Biotecnología y Genómica de Plantas, Universidad Politécnica de Madrid – Instituto Nacional de Investigación y Tecnología Agraria y AlimentariaMadrid, Spain; ^3^Department of Biology, Graduate School of Science, Kobe UniversityKobe, Japan

**Keywords:** Arabidopsis, root-knot nematodes, galls, leaf/callus, LBD16, *de novo* organogenesis, auxins, *Meloidogyne* spp

## Abstract

Root-knot nematodes (RKNs; *Meloidogyne* spp.) induce feeding cells (giant cells; GCs) inside a pseudo-organ (gall) from still unknown root cells. Understanding GCs ontogeny is essential to the basic knowledge of RKN–plant interaction and to discover novel and effective control strategies. Hence, we report for the first time in a model plant, Arabidopsis, molecular, and cellular features concerning ectopic *de novo* organogenesis of RKNs GCs in leaves. RKNs induce GCs in leaves with irregular shape, a reticulated cytosol, and fragmented vacuoles as GCs from roots. Leaf cells around the nematode enter G2-M shown by *ProCycB1;1:CycB1;1(NT)-GUS* expression, consistent to multinucleated GCs. In addition, GCs nuclei present irregular and varied sizes. All these characteristics mentioned, being equivalent to GCs in root-galls. RKNs complete their life cycle forming a gall/callus-like structure in the leaf vascular tissues resembling auxin-induced callus with an auxin-response maxima, indicated by high expression of *DR5::GUS* that is dependent on leaf auxin-transport. Notably, induction of leaves calli/GCs requires molecular components from roots crucial for lateral roots (LRs), auxin-induced callus and root-gall formation, i.e., LBD16. Hence, LBD16 is a xylem pole pericycle specific and local marker in LR primordia unexpectedly induced locally in the vascular tissue of leaves after RKN infection. LBD16 is also fundamental for feeding site formation as RKNs could not stablish in *35S::LBD16-SRDX* leaves, and likely it is also a conserved molecular hub between biotic and developmental signals in Arabidopsis either in roots or leaves. Moreover, RKNs induce the ectopic development of roots from leaf and root-galls, also formed in mutants compromised in LR formation, *arf7/arf19*, *slr*, and *alf4*. Therefore, nematodes must target molecular signatures to induce post-embryogenic *de novo* organogenesis through the *LBD16* callus formation pathway partially different from those prevalent during normal LR development.

## Introduction

Root-knot nematodes (RKNs; *Meloidogyne* spp.) are a major group of plant endoparasitic nematodes that cause vast economic losses in agriculture worldwide (Escobar et al., 2015). *Meloidogyne* spp. establishes a highly complex relation with the host plant at the cellular and molecular level by inducing inside the roots their own feeding cells (giant cells, GCs), as the only source of nutrients and prerequisite for the nematode parasitism within the plant. RKNs subtly migrate intercellularly through the vascular cylinder and use effectors to reset molecular pathways defined in root cells, inducing the development of feeding cells. It is documented that the nematode secretions contain phytohormones, such as auxins and cytokinins, and small peptides that could interfere with basic developmental pathways (reviewed in Cabrera et al., 2015a; Guo et al., 2017), leading to the hypertrophy and/or hyperplasia of cells in the vascular cylinder, endodermis and cortex that form a knot or gall, which contains the GCs. In the last years, investigations on the putative GCs precursor or stem root cells have led to the identification of specific molecular signatures contributing to the development of the GCs and galls from different root cell types, e.g., those from xylem pole pericycle, lateral root primordia (LRP), root apical meristem, or protoxylem (reviewed in Cabrera et al., 2015a).

Galls could be considered as a *de novo* originated pseudo-organ in roots with similarities with the formation of post-embryogenic organs, e.g., LRs, adventitious roots (ARs) or nodules induced by rhizobia. Hence, similar molecular components to those processes were identified in galls, such as crucial transcription factors (*PHAN*, *KNOX*, *LBD16*, *WRKY23;* Koltai and Bird, 2000; Grunewald et al., 2008; Cabrera et al., 2014) as well as the early nodulin gene *ENOD40* and the cell cycle control gene *CCS52a* in the case of rhizobia (Koltai et al., 2001; Favery et al., 2002). The transcriptomes of laser-micro-dissected GCs and galls at early infection stages (3dpi) were also similar to that of specific transcript profiles from LRP cells (Cabrera et al., 2014). Additionally, an auxin response maxima and a group of proliferating cells are also common among these processes. Recently, a clear link between nematode B-type CLE signaling and the TDIF-TDR (TDIF receptor)-WOX4 pathway which promotes procambial meristem cell proliferation, was described during beet cyst nematode *Heterodera schachtii* parasitism (Guo et al., 2017). In this respect, understanding how the RKNs interfere with basic developmental pathways leading to proliferation of cells and differentiation of new root cell types, should be a prerequisite to design new effective biotechnological tools for their control.

How plants generate new organs from post-embryogenic tissues is still an interesting challenge. In this respect, a crucial study determined that a callus, a pluripotent cell mass, resembles the tip of a root meristem, and a common mechanism in callus formation from disparate organs such as petals, cotyledons or roots is the ectopic activation of a lateral root (LR) development program (Sugimoto et al., 2010). Arabidopsis LATERAL ORGAN BOUNDARIES DOMAIN (LBD)/ASYMMETRIC LEAVES2-LIKE (ASL) transcription factors are common to the control of LR development and callus formation program; as ectopic expression of LBD genes in Arabidopsis is sufficient to trigger spontaneous callus formation without exogenous phytohormones, whereas suppression of its function inhibits callus formation triggered by auxins (Okushima et al., 2007; Fan et al., 2012). This indicates that LBDs/ASLs are key regulators of callus formation (Perianez-Rodriguez et al., 2014). Hormone induced *calli* are routinely used *in vitro* to subsequently regenerate different plant organs, i.e., shoots and roots by changing the auxin/cytokinin balance (Skoog and Miller, 1957). Interestingly, LBD16 through auxin signaling is induced and crucial for root-gall formation where a population of proliferative cells were also described (Cabrera et al., 2014; Escobar et al., 2015). However, little is known yet on the molecular mechanisms underpinning gall formation by different pathogenic organisms (Ikeuchi et al., 2013).

Here we report molecular and cellular aspects concerning the ectopic *de novo* organogenesis of RKNs feeding sites into the vascular tissues of Arabidopsis leaves. Our results show remarkable molecular and cellular parallelisms with the formation of galls in roots as well as hormone induced calli, LRs and ARs. We identified that LBD16 might be acting as a molecular switch between those developmental and environmental biotic (RKNs) signals triggering cell proliferation and/or formation of a new organ. Furthermore, RKNs induce the new formation of roots from their feeding sites in wild type plants and in mutants severely compromised in LR formation, confirming that to induce *de novo* organogenesis, nematodes should target molecular components, at least, partially different from those operating during normal LR development. The experimental system used reveals the enormous morphogenetic plasticity of plants in response to biotic environmental signals.

## Materials and Methods

### Plant Material and Nematode Population

*Arabidopsis thaliana* (L.) Heynh Columbia-0 (Col-0) was the background of all the transgenic lines. For expression analysis, we used *DR5::GUS* (Ulmasov et al., 1997), *pLBD16::GUS* in which *GUS* is expressed under the control of the 2.5 kb promoter sequence of *LBD16* (Okushima et al., 2007) and *ProCycB1;1:CycB1;1(NT)-GUS* (Colón-Carmona et al., 1999). The *alf4* mutant (Celenza et al., 1995) and the *pLBD16::GUS* line crossed to the *slr* and *arf7/arf19* mutants were used for LR emergence analysis.

Maintenance of the *Meloidogyne javanica* population *in vitro* on cucumber (*Cucumis sativus* cv Hoffmanns Giganta) and egg masses hatching was performed according to Díaz-Manzano et al. (2016).

### Growth Conditions and Nematode Inoculation

*Arabidopsis thaliana* Col-0 seeds were surface sterilized and sown in modified Gamborg B5 medium supplemented with 1.5% sucrose (GB5M1.5%; Gamborg et al., 1968). Plants were grown for 12 days at 23°C and a photoperiod of 16 h light/8 h dark. Around 800 freshly hatched juveniles in water were mixed in a proportion 1:6 with temperate modified Gamborg B5 medium with 3% sucrose (GB5M3%; Gamborg et al., 1968), pouring at least 2 mL into a well of a Falcon Tissue Culture Plates 6-well (Fisher-Scientific; Hampton, NH, United States). Fifteen leaves of each line were cut (Chen et al., 2014) and deposited in a well containing this modified GB5M3% with or without the addition of nematodes or chemical treatments [0.1–1 μM indole-3-acetic acid (IAA) or 1 μM naphthylphthalamic acid (NPA)] were added to the medium before the solidification. For each analysis, three independent experiments were performed with at least 15 leaves per line each. The plates were transferred to a chamber at 23°C, 70% humidity and total darkness.

The *alf4* mutant (Celenza et al., 1995) and the *pLBD16::GUS* line crossed to the *slr* and *arf7/arf19* mutants were grown and inoculated according to Olmo et al. (2017).

### Histological Analysis with Conventional Microscopy

For *GUS* staining, leaves were incubated for 2–8h in a solution containing 5 mM EDTA (pH 8), 0.05% Triton X-100, 0.5 mM K_3_Fe(CN)_6_, 0.5 mM K_4_Fe(CN)_6_, and 1 mg ml^-1^ X-GlcA in 50mM sodium phosphate buffer. After *GUS* staining the leaves were fixed in 3% glutaraldehyde for 15 minutes under moderate vacuum at room temperature and overnight at 4°C. Then, they were dehydrated in solutions of 30% ethanol (EtOH) for 20 min, 50% EtOH for 20 min and 70% EtOH overnight, followed by 90% EtOH for 1h and finally clarified for 2 days in a solution of chloralhydrate:glycerol:water (8:1:2 w/v/v). Leaves were mounted in the same solution in slides and photographed under a LeicaMz125 stereomicroscope (Leica-Microsystems, Wetzlar, Germany) or a Nikon eclipse 90i microscope (Nikon Corp., Tokyo, Japan). For tissue ultrastructural analysis, leaves were fixed, imbibed in araldite and sectioned at 2 μm as in Barcala et al. (2010). Galls in the roots were hand-sectioned and observed and photographed under a LeicaMz125 stereomicroscope (Leica-Microsystems, Wetzlar, Germany).

### Histological Analysis with Confocal Microscopy

For confocal microscopy, leaves were fixed in 3% glutaraldehyde for 15 min under moderate vacuum at room temperature and overnight at 4°C. Then, they were dehydrated in solutions of 30% EtOH for 20 min, 50% EtOH for 20 min, 70% EtOH for 20 min, 90% EtOH for 20 min, and finally 100% EtOH overnight. For clarification, leaves were incubated for 20 min in a solution 1:1 of EtOH:BABB [BABB: benzyl alcohol (Sigma 402834)/ benzyl benzoate (Sigma B6630) 1:2] and then incubated in 100% BABB for at least 20 min before observation. Leaves were mounted in BABB in slides and observed under Leica TCS SP2 confocal laser scanning microscope. Some leaves clarified with chloralhydrate were stained with 0.5 g/ml propidium iodide (PI) in phosphate-buffered saline (PBS) for 5 min while in those clarified with BABB, auto-fluorescence of tissues after glutaraldehyde treatment was used to observe the structures.

## Results and Discussion

The process of galls and GCs differentiation in which vascular root cells suffer massive molecular changes (e.g., in Arabidopsis, Jammes et al., 2005; Barcala et al., 2010) to drive dramatic morphogenetic events leading to the formation of a pseudo-organ (gall) and of feeding cells (GCs), is still scarcely known. However, the implication of cells from the xylem poles pericycle and molecular transducers, i.e., LBD16, relevant for LR development, was confirmed crucial for galls/GCs development in roots (Cabrera et al., 2014). Galls/GCs ontogeny is certainly rather unexplored. Hence, studying ectopic development of GCs by RKNs in the model system of Arabidopsis leaves could represent an advance in the identification of precursor cells and genetic pathways necessary for the development of nematode feeding sites elsewhere within the plant. In this respect, specific molecular pathways for GCs development might occur in addition or alternative to transduction pathways related to leaf development and/or maintenance. With this aim, we developed a system of study based on *Meloidogyne javanica* infection of Arabidopsis leaves. Hence, we checked whether galls/GCs were developed in leaves as they are in roots and studied molecular cues that might be conserved in leaves as compared to roots. We adapted a protocol for culturing excised Arabidopsis leaves *in vitro* upon RKN infection based on Liu et al. (2014), who used it for the study of AR formation. Furthermore, we also developed a method to clarify RKN feeding sites from roots and leaves that allow the direct observation of GCs, even at late infection stages and preserving subcellular structures, with no need of tedious hand sectioning, by confocal microscopy. With this method, we compared cytological features from GCs induced by RKNs either in leaves or roots.

The infection of aerial plant tissues by *Meloidogyne* spp. and other plant parasitic nematodes has been reported in several crop species (Linford, 1941; Powell and Moore, 1961; Shepperson and Jordan, 1968; Wong and Willetts, 1969; reviewed in Bird, 1974; Lehman and MacGowan, 1986; Cabrera Poch et al., 2006). However, it has never been reported in a model plant such as Arabidopsis.

### GCs and Feeding Sites Are Formed in Vascular Tissues of Leaves As Well As in Roots Showing Similar Cytological Features

**FIGURE 1:**
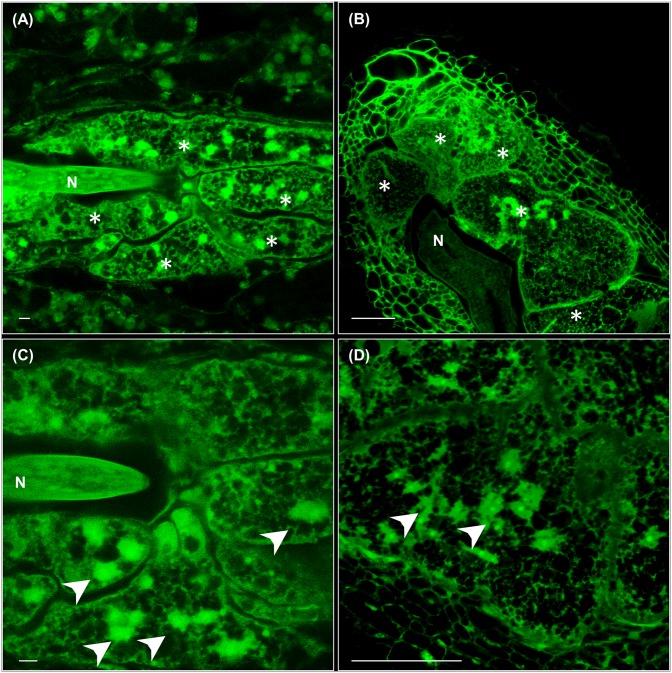
**Cytological features of feeding sites and giant cells (GCs) formed in leaves and in vascular root tissues by *Meloidogyne javanica*. (A)** A representative feeding site formed in a secondary vein of an Arabidopsis leaf at medium-late infection stage (30–40 days after inoculation). The anterior part of the nematode body is clearly in the middle of 4–5 GCs that are irregular in shape with a large size. **(B)** A representative feeding site formed in the roots of Arabidopsis at medium-late infection stage (21 days after infection). A large GC size with reticulated cytosols and many irregular, some clustered, nuclei are observed similarly either in GCs formed in leaves **(C)** or roots **(D)**. Scale bars: 50 μm. N, nematode. Asterisks, GCs. Arrow heads point to some nuclei.

By using the method of clarification described (see Materials and Methods) and by taking advantage of the auto-fluorescence of tissues after glutaraldehyde fixation, we could observe directly with confocal microcopy and no sectioning that the feeding sites induced by *M. javanica* either in leaves or roots were histologically equivalent at medium-late stages of infection. They contain 4–8 GCs surrounding the nematode head in a central position (**Figures 1A,B**) that showed a considerably increased volume as compared to the surrounding cells, irregular shape, and reticulated cytoplasm with no evident or fragmented vacuoles (**Figures 1C,D**). GCs from leaves were multinucleated with nuclei of varied sizes, some of them big and clustered as in GCs from galls formed within root tissues, also consistent with former descriptions (**Figure 1**; de Almeida-Engler and Gheysen, 2013).

### *Meloidogyne javanica* RKNs Complete Its Life Cycle in *Arabidopsis thaliana* Leaves

**FIGURE 2:**
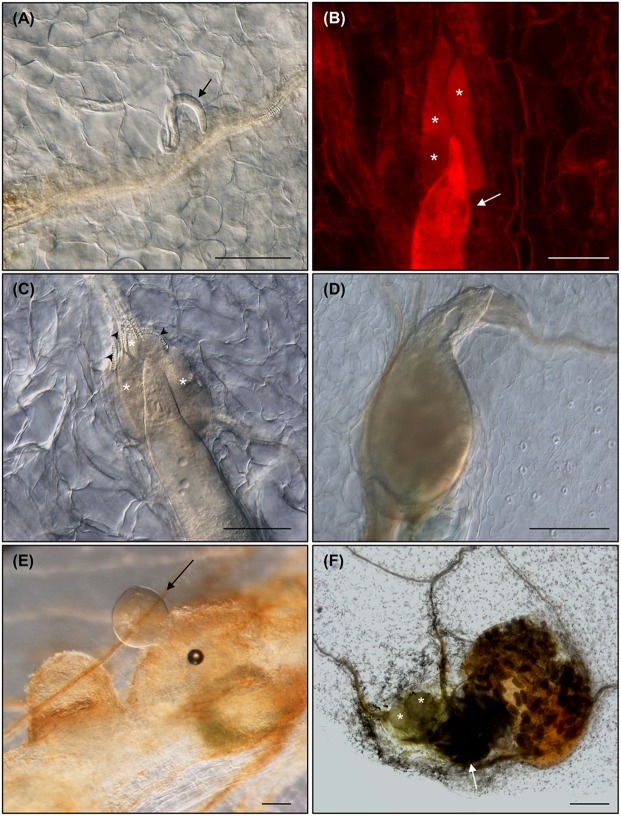
***Meloidogyne javanica* root-knot nematodes (RKNs) are able to complete their life cycle in *Arabidopsis thaliana* leaves. (A)** Nematode juvenile migrating through the parenchyma cells within a Col-0 leaf at 10 days after leaf culture and inoculation (daci), **(B,C)** established nematode feeding from clearly visible GCs at 18 and 24 daci, respectively (red fluorescence from propidium iodide staining), **(D,E)** developed females at 43 and 40 daci, respectively, and **(F)** life cycle was completed within excised leaves as shown by females with egg masses at 50 daci. Abundant xylem vessels around the GCs are also indicated with black arrow heads. Arrows and asterisks indicate the nematode and the GCs, respectively. *N* ≥ 45. Scale Bars: 100 μm **(A)** and 200 μm **(B–F)**.

**FIGURE 3:**
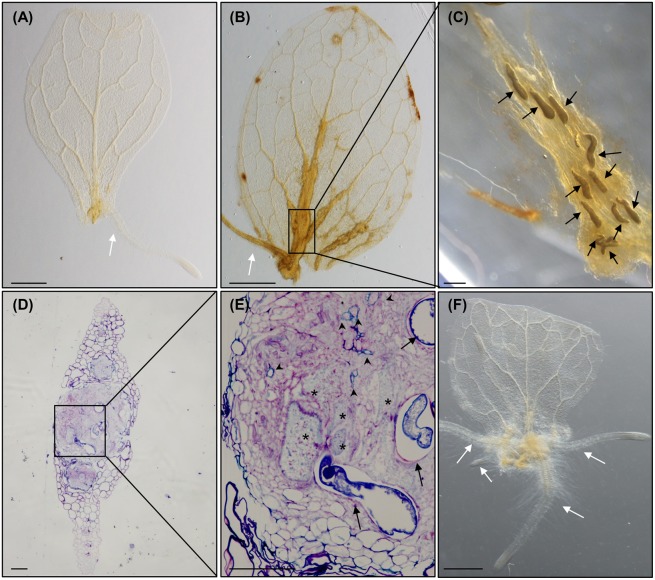
***Meloidogyne javanica* RKNs form a callus-like structure in *Arabidopsis thaliana* leaves resembling calli formed with exogenous auxins. (A)** Arabidopsis leaves cultured in Gamborg B5 medium supplemented with 3% sucrose, in the absence of any additional phytohormones, at 8 days after culture (dac). **(B)** Arabidopsis leaves cultured in Gamborg B5 medium supplemented with 3% sucrose, in the absence of any additional phytohormones but inoculated with *M. javanica* juveniles, at 18 daci, callus-like structures are observed along the veins. **(C)** Close up view of the nematodes established inside the callus- like structure induced in an infected leaf embedded in araldite at stages J2 to J4, **(D,E)** semithin sections of these leaves stained with toluidine in the area of the callus-like structures where GCs are clearly observed close to the female. **(F)** Leaf explant when cultured in Gamborg B5 implemented with 3% sucrose containing 0.1 μM of exogenous IAA at 8 dac. Black arrows, white arrows and asterisks indicate the nematode, the adventitious roots (ARs) and the GCs, respectively. Abundant xylem vessels around the GCs are also indicated with arrow heads in **E**. *N* ≥ 45. Scale Bars: 500 μm **(A,B,F)** and 100 μm **(C–E)**.

**FIGURE 4:**
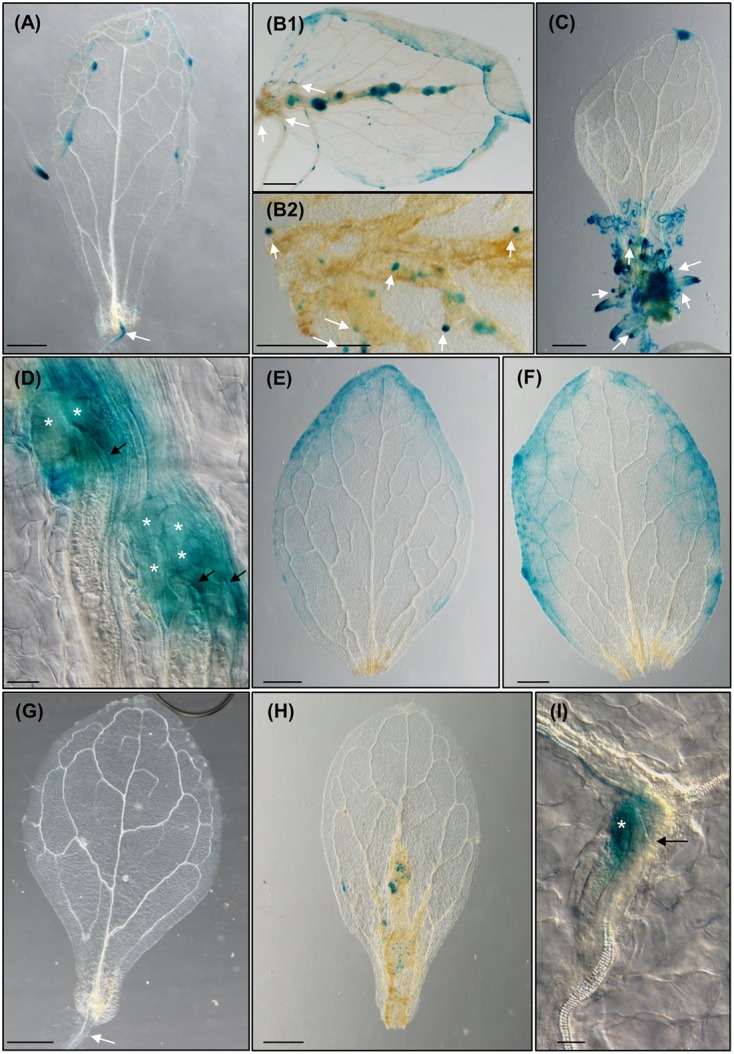
**The formation of *Meloidogyne javanica* induced callus in *Arabidopsis thaliana* leaves is dependent on the accumulation of auxins transported from the leaf and activates G2-M cell cycle transition. (A)** Arabidopsis leaves from the reporter line *DR5::GUS* cultured in Gamborg B5 medium supplemented with 3% sucrose, in the absence of any additional phytohormones, at 8 dac. **(B)** Arabidopsis leaves from the reporter line *DR5::GUS* cultured in Gamborg B5 medium supplemented with 3% sucrose, in the absence of any additional phytohormones but inoculated with *M. javanica* juveniles, at 10 and 12 dac and inoculation (daci; B1 and B2, respectively). Numerous growing callus-like structures along the vascular cylinder express GUS; emerging ARs primordia expressing GUS from the callus-like structures are also observed (B2). **(C)** *DR5::GUS* leaf explant when cultured in Gamborg B5 implemented with 3% sucrose containing 0.1 μM of exogenous IAA at 8 dac, calli in the excision area and emerging ARs observed express GUS. **(D)** Close up view of the nematode feeding cells in leaves treated as in **(B)**. *DR5::GUS* leaf explants uninfected **(E)** or infected **(F)** by nematodes when cultured in Gamborg B5 implemented with 3% sucrose containing 1 μM of NPA at 8 dac/daci; no calli neither ARs are observed. **(G)** Arabidopsis leaves from the reporter line *ProCycB1;1:CycB1;1(NT)-GUS* cultured in Gamborg B5 medium supplemented with 3% sucrose, in the absence of any additional phytohormones, at 8 dac. **(H)** Arabidopsis leaves and **(I)** close-up view from the reporter line *ProCycB1;1:CycB1;1(NT)-GUS* cultured in Gamborg B5 medium supplemented with 3% sucrose, in the absence of any additional phytohormones but inoculated with *M. javanica* juveniles, at 8 and 10 daci, respectively; groups of cells expressing GUS indicative of cells entering G2-M are clearly observed. Black arrows, white arrows and white asterisks indicate the nematode, the ARs and the GCs, respectively. *N* ≥ 45. Scale Bars: 500 μm **(A–C,E–H)** and 100 μm **(D)**.

Arabidopsis Col-0 seedlings were grown for twelve days in Gamborg B5 medium supplemented with 1.5% sucrose (GB5M1.5%; Gamborg et al., 1968). The first true leaves were cut at the region between the blade and petiole and sub-cultured in the dark in Gamborg B5 medium supplemented with 3% sucrose (GB5M3%; Chen et al., 2014) containing approximately 800 *M. javanica* juveniles. Ten days after culture and inoculation (daci), several nematode juveniles could be observed migrating through the parenchyma cells inside the leaves (**Figure 2A**). Visualization procedure requires dehydration with increased ethanol series and clarification in a solution of chloralhydrate:glycerol:water (8:1:2 w/v/v) for at least 2 days, thus chlorophylls are not present. No damaged cells were observed in the leaf epidermis (**Figures 2A–C**), neither in other leaf tissues, apart from those produced while cutting the petiole-blade junction. This indicates that RKNs penetrate and migrate in the leaves intercellularly as in roots. Therefore, migratory habits of RKNs are maintained in plant parts other than roots, similarly to the behavior of the cyst nematodes that cause cell death during migration in roots and in the leaves (Cabrera Poch et al., 2006; Bohlmann, 2015). After the nematode is established, GCs associated to the nematodes started to develop at 18 or 24 daci (**Figures 2B,C**). As in root infections (reviewed in Escobar et al., 2015), new vascularization around the GCs was clearly observed in leaves as profuse xylem vessels were present around the GCs (**Figures 2C**, **3E**). By 40 or 43 daci, the nematodes had completed their life cycle inside the leaves (**Figures 2D,E**) and by 50 daci the female had deposited the egg mass (**Figure 2F**). Nematodes in different developmental stages were observed at the same daci, indicating the existence of a higher asynchrony in the nematode invasion/migration and establishment in leaf infections than in roots. Most nematode infections were associated to the central vein close to the cutting area. However, established nematodes could also be observed along secondary veins in other areas of the leaf (e.g., **Figures 3B**, **4B2**, **5C**). Hence, we confirmed that RKNs fully complete their life cycle with egg masses deposition in Arabidopsis *in vitro* grown leaves, thus the GCs formed were fully functional.

**FIGURE 5:**
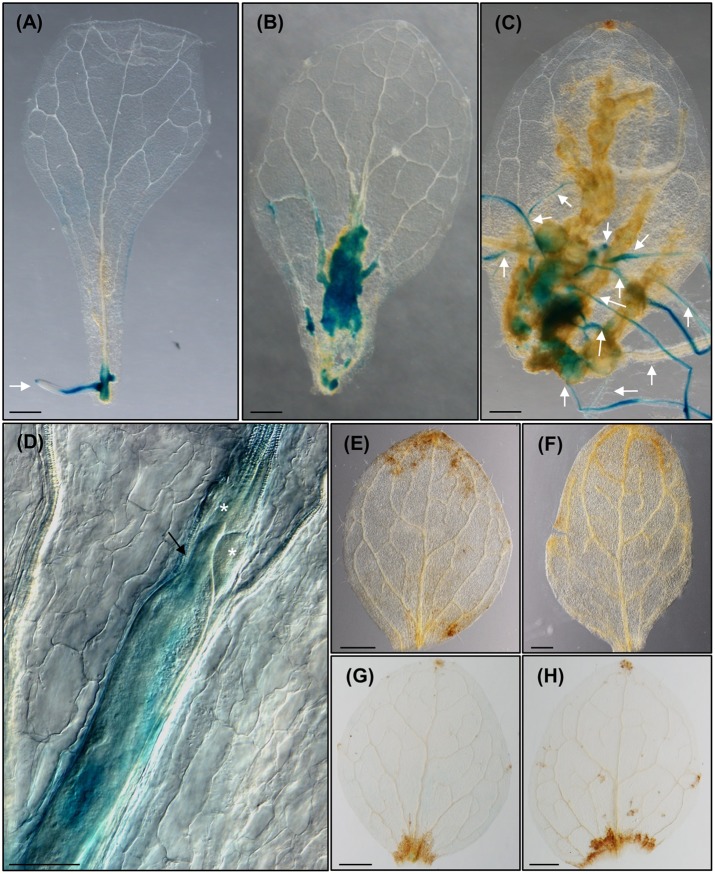
***Meloidogyne javanica* infection in leaves activates *LBD16*, a molecular marker of roots expressed in the xylem pole pericycle in LRP that is also crucial for feeding site formation in leaves. (A)** *pLBD16::GUS* leaf explants cultured in Gamborg B5 medium supplemented with 3% sucrose, in the absence of any additional phytohormones at 8 dac; GUS is observed in primordia and emerged ARs. **(B–D)** Leaves infected by nematodes at 10 **(B)**, 24 **(C)**, and 16 **(D)** daci, GUS signal is intense in callus-like induced by RKNs and ARs emerging from primary and secondary veins. **(E)** *35S::LBD16-SRDX* leaf explants uninfected or **(F)** infected by nematodes when cultured in Gamborg B5 implemented with 3% sucrose at 8 dac/daci. **(G,H)** *pLBD16::GUS* leaf explants uninfected **(G)** or infected **(H)** by nematodes when cultured in Gamborg B5 implemented with 3% sucrose containing 1 μM of NPA at 8 and 12 dac/daci, respectively. Nematodes cannot establish in *35S::LBD16-SRDX* plants nor in NPA treated leaves. White arrows, white arrow heads, black arrows and asterisks indicate the ARs, the galls, the nematode and the GCs, respectively. *N* ≥ 45. Scale Bars: 500 μm **(A–C,E–H)** and 200 μm **(D)**.

### *Meloidogyne javanica* Forms a Callus-Like Structure in *Arabidopsis thaliana* Leaves Resembling Auxin-Induced Callus

**FIGURE 6:**
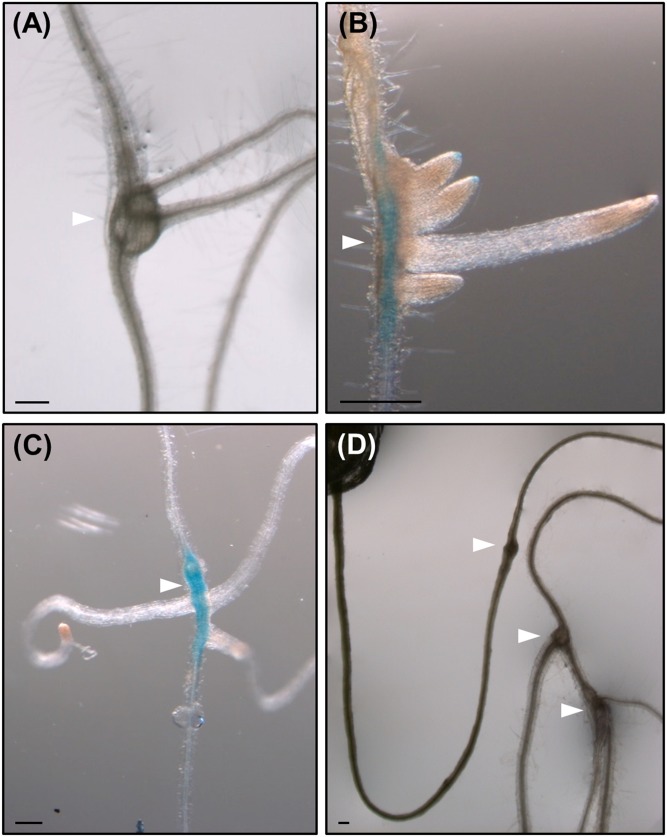
**Root-knot nematodes galls induced in roots of lateral root (LR) impaired mutant lines, show *de novo* organogenesis of non-canonical LRs as well as in wild type plants.** Emergence of LRs-like structures with no alternate pattern from galls induced by *M. javanica* in Col 0 **(A)**, in *arf7/arf19* **(B)**, and *slr* **(C)** mutants expressing *pLBD16::GUS*, and in *alf4* **(D)** mutant. White arrow heads indicate the galls. Scale Bars: 200 μm.

It has been demonstrated that Arabidopsis leaves cultured in the dark in GB5M3%, and in the absence of supplemented phytohormones, develop ARs from the vascular cylinder in the cutting area which are visible from 6 to 12 days after culture (dac). No new ARs are formed after 12 dac. These ARs appear to originate from procambial tissues forming primordia that finally develop into post-embryogenic roots without any other apparent change in leaf morphology (**Figure 3A**; Chen et al., 2014; Liu et al., 2014). In those leaves inoculated with nematodes, a mass of proliferative cells, which resembled a callus-like structure, developed from the cutting area upwards following the veins of the leaf that was clearly pronounced at 18 daci (**Figure 3B**). Only once the callus-like structure was formed, ARs then started to develop from this structure (**Figures 3B, 4B1,B2, 5C**). The fact that nematodes form first a callus-like structure, from which ectopic ARs are formed, suggests that infection may exploit or interfere with the normal developmental mechanism used for postembryonic root organogenesis. Similarly, we also observed ectopic LRs or ARs formed from the galls developed in the roots (**Figure 6A**).

Several nematodes in the J2–J4 stages were observed inside the callus-like structure in the leaves (**Figure 3C**). Semithin sections of these nematode-induced callus showed a high proportion of infection sites with developed GCs (**Figures 3D,E**). Tissues around the GCs proliferated, presenting a disorganized structure, and both, the GCs and the proliferated tissue, showed differential toluidine-cell wall staining as compared to the rest of the leaf cells. This might be due to thinner cell walls and/or different composition (**Figures 3D,E**), which could be consistent with molecular changes observed in the transcriptome of GCs from root-galls showing that secondary metabolism genes related to lignin deposition were repressed (Barcala et al., 2010; Portillo et al., 2013).

The formation of this callus-like structure and the development of ARs (**Figures 3B, 4B1,B2, 5C**), is similar to what happens to the leaf explants when cultured in GB5M3% sucrose supplemented with 0.1 and 1 μM of exogenous natural auxin (IAA) (**Figure 3F**; Liu et al., 2014). Liu et al. (2014) demonstrated that the formation of callus or ARs in leaves explants is dependent on the concentration of auxin in the medium. Hence, the infection of the leaves by nematodes, which results in the formation of callus, suggests that the presence of nematodes in the cutting area, may rely in local increment of the auxin concentration, probably triggering formation of callus. The presence of an auxin-response maxima in root galls as a consequence of RKNs infection, was solidly described (Hutangura et al., 1999; Absmanner et al., 2013; Cabrera et al., 2014) based on molecular reporters such as *DR5::GUS* (Ulmasov et al., 1997). Our results show a putative parallelism between the formation of the nematode feeding sites in leaves and IAA-induced callus formation in Arabidopsis which in turn may be triggered by changes in phytohormone balance, such as an increase in auxin concentration.

### The Formation of the RKN-Induced Callus in *Arabidopsis thaliana* Leaves Is Dependent on the Accumulation of Auxins Transported from the Leaf

To compare and decipher the putative role of auxins in the two morphogenic processes triggered in the leaf (i.e., exogenous auxin-induced calli and RKN-induced feeding sites resembling calli), we treated excised leaves from the reporter line *DR5::GUS*, with either nematodes or exogenous auxins (**Figure 4**). In those control untreated leaves, *GUS* expression was hardly detected in the cutting area, but clearly detected in the ARs primordia, and no callus had developed (**Figure 4A**). In contrast, leaves treated with IAA (**Figure 4C**) showed a strong *GUS* expression in the callus structures formed in the excision zone and in the primordia of ARs. Those leaves inoculated with nematodes showed a strong GUS signal in the callus-like structures formed in the excision zone and along the vascular cylinder where numerous growing callus-like structures expressed GUS too (**Figure4B1**). Numerous ARs were also observed emerging from those callus-like structures either from the main vein or from secondary veins (**Figures4B2**, **5C**). The GCs induced by *M. javanica* immersed in those calli also showed strong GUS signal (**Figure 4D**). These results reinforce the idea that the nematodes manipulate auxin levels or their response to create maxima within the vascular tissues of the leaf. Moreover, in untreated leaves, this auxin response was not observed (**Figure 4A**). It is possible that the presence of the nematodes in leaf zones showing high DR5 response is due to increments in auxin concentration. To further study the dependency of the plant auxin polar transport to build these auxin response maxima, as described in root galls (Kyndt et al., 2016) and AR primordia formation in leaves (Liu et al., 2014), we performed experiments with the auxin polar transport inhibitor NPA. *DR5::GUS* leaf explants cultured on GB5M3% sucrose and containing 1 μM NPA did not develop primordia leading to ARs formation at 12 dac (**Figure 4E**) as previously described (Liu et al., 2014). Untreated controls formed ARs(**Figures 3A–****4A**). Similarly, leaves cultured with NPA and inoculated with nematodes did not develop the callus-like structure at 12daci (**Figure 4F**), presenting the same phenotype than the uninfected control leaves (**Figure 4E**). Occasionally, several nematodes penetrate into the leaves and try to establish without success (Supplementary Figure S1). The fact that the nematodes were observed inside the leaves but they could not establish, suggests that auxin polar transport through the leaf is necessary for the establishment and proper development of the nematode feeding sites and GCs and to the generation of the callus-like structure that hosts them also in the leaves.

### RKN Induced Leaf Calli Share Molecular Components Crucial for Gall Formation in Roots

The reporter line *ProCycB1;1:CycB1;1(NT)-GUS*, which label only those cells that are entering the G2/M phase of the cell cycle (Colón-Carmona et al., 1999) was used to check active divisions within those RKN-induced callus-like structures. At 8 dac, *GUS* expression was not observable in the uninfected leaf explants (**Figure 4G**), except for that in the new AR primordia (data not shown), however, a specific signal was observed in the infected leaves in numerous cells within the calli along the vascular cylinder (**Figure 4H**). Visible signal was centered in those cells close to the nematode head inside the callus-like structure (**Figure 4I**). A similar pattern was observed in root galls in the initial divisions of the xylem pole pericycle cells around the nematodes head, e.g., at 3 days post infection (dpi). As the infection progresses, *ProCycB1;1:CycB1;1(NT)-GUS* expression spreads to small cells that fill the vascular cylinder inside the gall (Cabrera et al., 2014).

To check whether *LBD16* is induced in the feeding sites formed within the leaves, similarly to what occurs in galls from roots, we inoculated leaf explants from the *pLBD16::GUS* reporter line (Cabrera et al., 2014), with nematode juveniles. *LBD16* expression in the uninfected leaves was only observable in the new ARs and ARs primordia at 8 dac (**Figure 5A**), while a specific induction of the *pLBD16::GUS* was observed in early stages of the nematode-induced callus-like structures formation. Some of them were formed in the basal region of the leaf and others in the middle part of the leaves (10 daci; **Figure 5B**). In late infection stages, at 24 daci the expression of the *pLBD16::GUS* was lower and scattered throughout the callus-like structure, probably due to different stages of development of nematode infection found within the same leaf (**Figure 5C**). Interestingly, *pLBD16::GUS* expression is also clearly detected in emerging ARs coming from the nematode-induced calli located in secondary veins, a process not observed in auxin induced calli (compare **Figures4B2**, **5C** to **Figures 3F**, **4C**). At 24 daci, the callus-like structure formed in the infected leaves is as pronounced (**Figure 5C**) as those induced in leaf explants treated with exogenous auxins (**Figure 3F**), although it extends to most leaf areas and it is not confined to the cutting area. Moreover, a clear signal is also observed within the GCs induced by the nematode in the leaves at 16daci, thus *pLBD16::GUS* is also induced in GCs from leaves (**Figure 5D**). The expression pattern of *LBD16* resembles that obtained in root galls, where the *pLBD16::GUS* is activated at the early infection stages of 3 and 7 dpi but is hardly detectable at 21 dpi (Cabrera et al., 2014). The high asynchrony observed in the penetration and establishment in the leaves by RKNs, much more pronounced than in root infections, precluded detailed temporal data on GUS induction kinetics in infected leaves. Surprisingly, the promoter of a xylem pole pericycle marker from LR primordia, *LBD16*, is active in nematode induced feeding sites in leaves, that closely resembles its activation in roots-galls.

Next, we analyzed whether LBD16 was involved in RKN infection in leaves. *M. javanica* could not establish in leaves of the *35S::LBD16-SRDX* lines and no evidence of nematode-induced calli or ARs were observed (**Figure 5F**), similar to the non-infected control (**Figure 5E**). This is also similar to the strong reduction of the number of root-galls developed in this line (Cabrera et al., 2014), though in leaves the establishment seems fully blocked.

LBD16 is a crucial transcription factor not only during the organogenesis of LRP (Okushima et al., 2007; Goh et al., 2012) and galls (Cabrera et al., 2014) but also during the formation of callus from plant explants (Fan et al., 2012), as overexpression of LBD16 and other LBDs could lead to the ectopic formation of callus not only in wild type roots but also in the *arf7/arf19* mutant (Fan et al., 2012). Furthermore, we confirmed that LBD16 is also crucial for the development of RKN induced-calli and therefore, for nematode establishment and GCs formation in leaves. The induction of *LBD16* in both nematode-induced calli in leaves and in exogenous auxin induced calli (Fan et al., 2012), in LRP (Laplaze et al., 2005) and in ARs (Welander et al., 2014), constitute a strong molecular link among these processes. Hence, the expression of *LBD16* in the nematode feeding sites in leaves without the participation of exogenous auxins and where no LRP are present, brings the idea that galls development could also be related to callus formation, and not only to LRP development as pointed for root-galls (Cabrera et al., 2014). *In silico* data comparison supported this hypothesis, as expression of co-regulated genes with *LBD16* in early developing GCs and galls was similar to those found in transcriptomes from root explants differentiating to callus (Cabrera et al., 2015b). Hence, LBD16 might be acting as a molecular switch between those developmental and environmental biotic (RKNs) signals triggering cell proliferation and/or formation of a new organ; revealing the enormous morphogenetic plasticity of plants in response to biotic environmental signals.

It has been demonstrated that the development of callus in Arabidopsis also requires several components of LR developmental pathways, including *LBDs* (Sugimoto et al., 2010; Fan et al., 2012; Perianez-Rodriguez et al., 2014). In nematode feeding sites formed in leaves, the expression pattern of *LBD16* resembles that observed in root galls and, hence, both should share genes that were described as clear markers of early initial stages of LR primordia (Laplaze et al., 2005). This suggests also a close relationship among LR formation and feeding site formation either in leaves or roots, that may be controlled by physiological and genetic modifiers, which in turn, may be used and exploited by nematodes to cause infection.

Interestingly, an important question raised by these observations is from which particular tissues the nematodes induce the galls or the callus-like structures in roots or leaves. In plants, the procambium or cambium is a meristematic tissue that contains cells maintaining or capable of acquiring stem cell characteristics in adult leaves (Lachaud et al., 1999; Sugimoto et al., 2010). In addition, it contains specific cell populations expressing markers shared with the pericycle such as J0121 (Dubrovsky et al., 2000; Sugimoto et al., 2010). Organogenesis of ARs and the formation of callus initiate from procambium cells expressing J0121 in excised aerial organs explants (Yu et al., 2010; Sugimoto et al., 2010; Chen et al., 2014). Thus, it is possible that nematode secretions manipulate those J0121 expressing cells or other remaining meristematic tissues in leaves and roots in a similar way to postembryonic organogenesis to differentiate their feeding sites. This is totally in agreement to the important role of the xylem pole pericycle cells during gall formation in roots as genetic ablation driven by a J0121 > > DTA line severely compromised RKNs infection (Cabrera et al., 2014). In addition, cell divisions in other tissues from roots and leaves, could be contributing to the formation of the galls. Accordingly, it has been recently demonstrated that the TDIF-TDR (TDIF receptor)-WOX4 pathway, which promotes procambial meristem cell proliferation, is involved in cyst nematode parasitism. Moreover, isolated A-type and B-type CLE peptides from *Heterodera schachtii*, induced massive cell proliferation in wild type roots, suggesting that the two types of CLEs may regulate cell proliferation during feeding site formation (Guo et al., 2017). Although, cyst nematodes feeding cells, are structurally different to that of GCs, as they form syncytia from fusion of adjacent cells, whereas RKNs form GCs from acytokinetic mitosis, this finding is relevant as CLE-like peptides were also identified from *Meloidogyne incognita* secretions and calli were observed in Arabidopsis and tobacco overexpressing 16D10 (a secretory peptide with a CLE-Like sequence; Huang et al., 2006).

Importantly, in excised *pLBD16::GUS* leaves cultured in the presence of 1 μM NPA and either uninfected (**Figure 5G**) or infected (**Figure 5H**) with nematodes, no *GUS* expression nor formation of callus and/or ARs was observed, similarly to that shown for the reporter line *DR5::GUS* (**Figures 4E,F**). As LBD16 can be activated downstream of auxin signaling, this reinforces the idea that an auxin response maximum in leaf callus-like structures is induced by nematodes through manipulation of auxin polar transport from distal parts of the leaf. This idea is in accordance to that observed in root-gall development (Kyndt et al., 2016). Organogenesis of primordia leading to AR formation in leaf explants, depends on auxin polar transport to form auxin maxima (Liu et al., 2014), while DR5 maxima oscillations necessary to initiate LRs may also require auxin transport (Moreno-Risueno et al., 2010; Xuan et al., 2015). Our data suggest that phytohormone imbalance leading to new auxin response maxima formation, might be a common primary trigger for post embryogenic organogenesis processes, such as LRP, ARs, calli and nematode feeding site formation (**Figures4**, **5**).

### RKN Induced Galls in Roots of LR Impaired Mutant Lines Show *De novo* Organogenesis of Non-Canonical LRs

**FIGURE 7:**
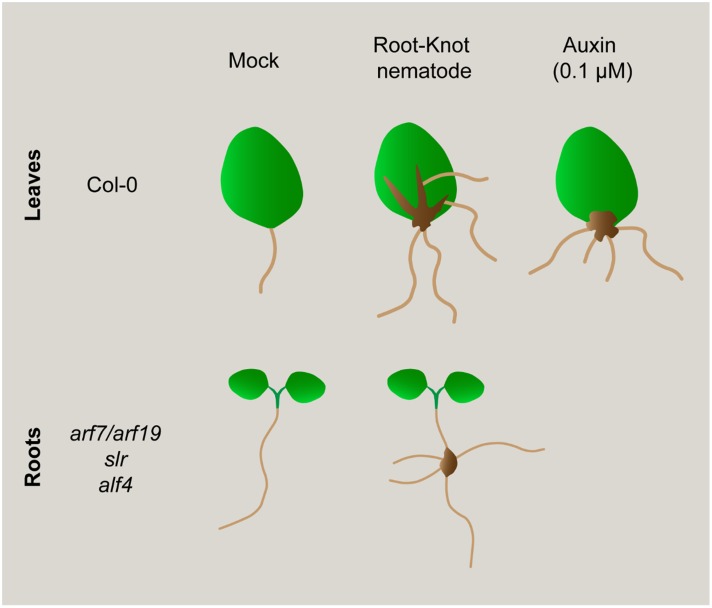
**Schematic representation of morphogenic processes induced in leaves after different treatments: Exogenous auxin induced roots and infection with a RKN.** Nematodes induce new root formation (ARs) from callus-like structures in the primary veins of leaves, similar to what happen from callus induced after exogenous auxin treatment. However, nematode induced callus-like also develop ARs from secondary veins, a process that does not occur after only auxin treatment. Interestingly, non-canonical roots are also induced in root-galls from mutants severely impaired in LR formation (i.e., *arf7/arf19*, *slr*, *alf4*). Hence, nematodes could induce new root formation in galls bypassing upstream key regulators for LR formation or by using partially different molecular pathways.

It has been observed that root-galls frequently contain LR primordia and many roots finally emerge from them frequently with no canonical left-right alternate pattern (**Figure 6A**); perhaps, induced by a process similar to that also forming ectopic ARs in the nematode induced-calli of leaves (**Figures3**, **4**, **5**). Surprisingly, when three of the most severe Arabidopsis mutants with no LR growth described were infected with RKNs, i.e., *slr, arf7/arf19*, and *alf4* (Celenza et al., 1995; Fukaki et al., 2002; Okushima et al., 2007), some of the formed root galls showed a profuse emergence of LR-like organs with atypical left-right pattern (**Figures 6B–D**). Therefore, nematodes induce new root formation in galls either by circumventing upstream key regulators for LR formation or by using different molecular pathways. In any case, our results show that specific molecular signatures must be targeted by the nematodes to induce post-embryogenic *de novo* organogenesis that can be partially different from those operating during normal LR development (**Figure 7**).

## Conclusion

Our data suggest that phytohormone imbalance leading to new auxin response maxima formation, could be a common primary trigger for post embryogenic organogenesis processes, such as LRs, ARs, calli, and nematode feeding site formation in roots and leaves. In this respect, the molecular cues of new organs generation from post-embryogenic tissues is still an intriguing challenge. Callus formation from disparate organs such as petals, cotyledons or roots activates commonly a LR development program (Sugimoto et al., 2010). We presented several characteristics of nematode feeding sites in leaves that resembles a calli, such as a mass of proliferating tissue with an auxin maxima response, but overall, we identified that LBD16 could be acting as a molecular hub between those developmental and environmental biotic (RKNs) signals triggering cell proliferation and/or formation of a new organ such as galls, calli and LR. Notably, RKNs induce the new formation of roots from their feeding sites in wild type plants and in mutants severely compromised in LR formation, confirming that to induce *de novo* organogenesis, nematodes should also target molecular components, at least partially different from those operating during normal LR development.

We also believe that our approach could contribute to find more common molecular switches to essential post-embryogenic organogenesis processes, i.e., LRs, ARs, and calli, including nematode-induced root galls and leaf calli (**Figure 7**). Finally, it brings to light the enormous morphogenetic plasticity of plants in response to biotic environmental signals such as RKN infection.

## Author Contributions

Conceptualization: RO, JC, and CE; Methodology: RO and JC; Investigation: RO and JC; Writing – Original Draft: RO, JC, and CE; Writing – Review and Editing: JC, MM-R, HF, CF, and CE; Funding Acquisition: CE and CF; Resources: CF and CE; Supervision: CE.

## Conflict of Interest Statement

The authors declare that the research was conducted in the absence of any commercial or financial relationships that could be construed as a potential conflict of interest.
