# Supplementary figures and images for: Molecular Transducers from Roots Are Triggered in Arabidopsis Leaves by Root-Knot Nematodes for Successful Feeding Site Formation: A Conserved Post-Embryogenic De novo Organogenesis Program?
Source: Front Plant Sci. 2017 May 26;8:875. doi: 10.3389/fpls.2017.00875 (PMC5445185; doi:10.3389/fpls.2017.00875)

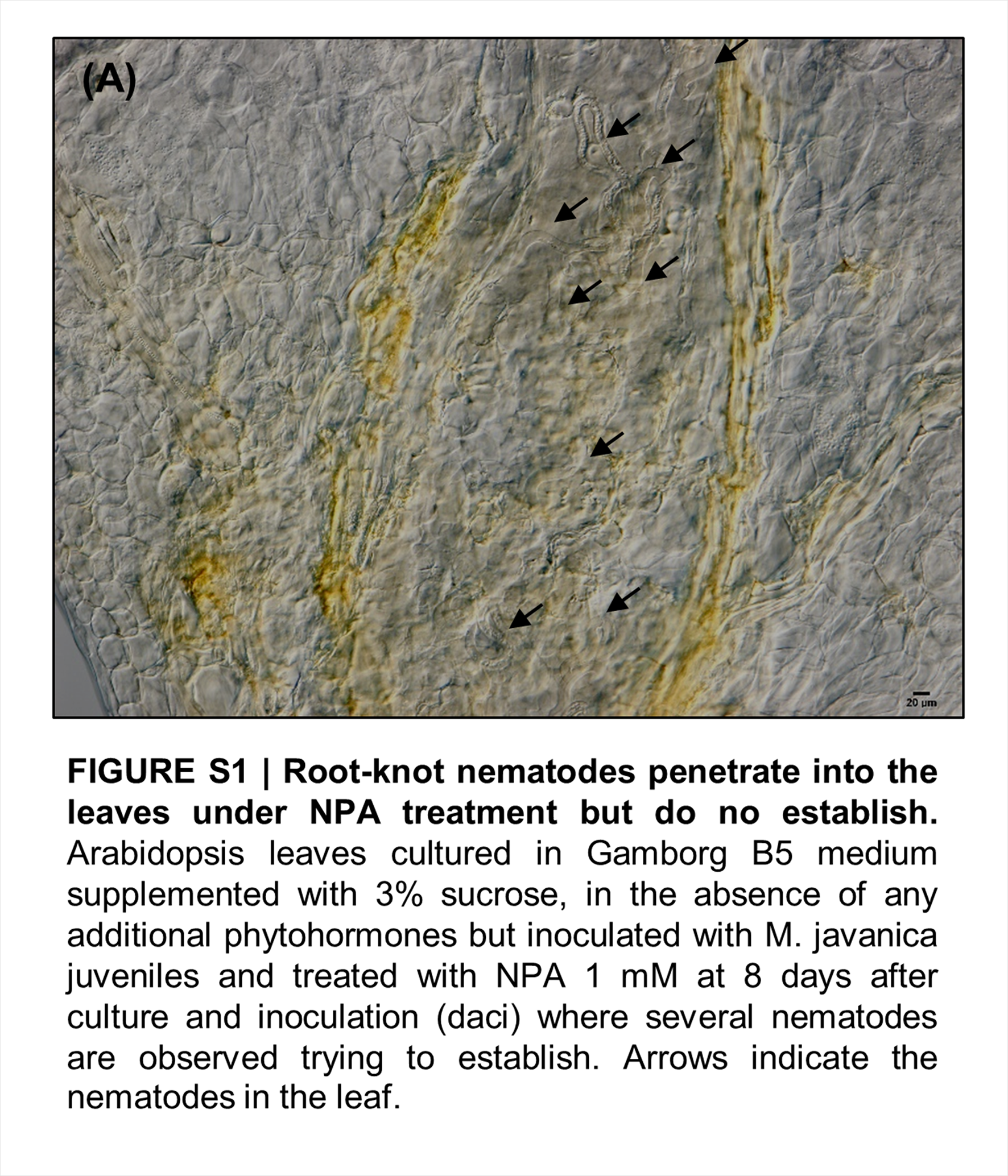

Supplement: Supplementary file 1 [file Image_1.tif]
